# The stress-vulnerability model on the path to schizophrenia: Interaction between BDNF methylation and schizotypy on the resting-state brain network

**DOI:** 10.1038/s41537-022-00258-4

**Published:** 2022-05-06

**Authors:** Hye Yoon Park, Minji Bang, Eunchong Seo, Se Jun Koo, Eun Lee, Seung-Koo Lee, Suk Kyoon An

**Affiliations:** 1 Department of Psychiatry, Yonsei University Wonju College of Medicine, Wonju, Republic of Korea; 2 Section of Self, Affect and Neuroscience, Institute of Behavioral Science in Medicine, Yonsei University College of Medicine, Seoul, Republic of Korea; 3 Department of Psychiatry, CHA Bundang Medical Center, CHA University, Seongnam, Republic of Korea; 4 Graduate Program in Cognitive Science, Yonsei University, Seoul, Republic of Korea; 5 Department of Psychiatry, Yonsei University College of Medicine, Severance Hospital, Seoul, Republic of Korea; 6 Department of Radiology, Yonsei University College of Medicine, Severance Hospital, Seoul, Republic of Korea

**Keywords:** Schizophrenia, Psychosis

## Abstract

The interplay between schizophrenia liability and environmental influences has been considered to be responsible for the development of schizophrenia. Recent neuroimaging studies have linked aberrant functional connectivity (FC) between the default-mode network (DMN) and the frontoparietal network (FPN) in the resting-state to the underlying neural mechanism of schizophrenia. By using schizotypy as the proxy for genetic-based liability to schizophrenia and methylation of brain-derived neurotrophic factor (BDNF) to represent environmental exposure, this study investigated the impact of the interaction between vulnerability and the environment on the neurobiological substrates of schizophrenia. Participants in this study included 101 healthy adults (HC) and 46 individuals with ultra-high risk for psychosis (UHR). All participants were tested at resting-state by functional magnetic resonance imaging, and group-independent component analysis was used to identify the DMN and the FPN. The Perceptual Aberration Scale (PAS) was used to evaluate the schizotypy level. The methylation status of BDNF was measured by pyrosequencing. For moderation analysis, the final sample consisted of 83 HC and 32 UHR individuals. UHR individuals showed reduced DMN-FPN network FC compared to healthy controls. PAS scores significantly moderated the relationship between the percentage of BDNF methylation and DMN-FPN network FC. The strength of the positive relationship between BDNF methylation and the network FC was reduced when the schizotypy level increased. These findings support the moderating role of schizotypy on the neurobiological mechanism of schizophrenia in conjunction with epigenetic changes.

## Introduction

Schizophrenia is a devastating mental illness that manifests early in life. The development trajectory of schizophrenia is heterogeneous but typically consists of a premorbid period with nonspecific developmental or behavioral deviances^1^, a prodromal phase with a subclinical presentation of illness from first noticeable symptoms to prominent psychotic symptoms^2,3^, and an active phase with the onset of a fully psychotic episode. Although the exact neuronal mechanisms of schizophrenia remain unknown, neuroimaging has found extensive altered functional connectivity (FC) patterns in resting-state brain networks^4^. Among disconnections across brain networks, reduced connectivity between the default-mode network [DMN; involved in internally oriented cognition and the self-related thought process^5^] and the frontoparietal network [FPN; involved in external task performance and goal-directed regulation^6^] has been consistently reported in individuals with schizophrenia [for meta-analysis, see Dong et al.^7^]. Furthermore, this aberrant coupling of DMN-FPN has also been demonstrated in individuals at ultra-high risk (UHR) for psychosis^8,9^ who are in the ‘putative’ prodromal phase of schizophrenia. Therefore, the abnormal coordination between DMN and FPN, which reflects dysregulation between a self-focused mode and stimulus-dependent attention^10^, may drive the path to schizophrenia, which is characterized by confusion between internal thoughts and external reality.

Schizophrenia liability and schizophrenia-like traits exist as part of continuum in the general population^11,12^. Schizotypy, a set of enduring traits that reflect the subclinical symptoms and signs of schizophrenia, has been proposed as a proxy for genetic-based liability to schizophrenia^13–15^ and is conjectured to be found in 10% of the general population^14^. Empirical work supports the commonality of schizotypy with schizophrenia in terms of shared genetics^12^ and abnormalities in cognition, including perceptual experiences across all sensory modalities^16,17^. Although the reports were highly heterogenous, resting-state functional neuroimaging studies showed comparable altered networks in individuals with high levels of schizotypy and schizophrenia^18–20^. Schizotypal symptoms have also been associated with FC in healthy adults^20^, individuals with schizotypal personality disorder^21^, and schizophrenic individuals^22^. Thereby, it is empirically supported that schizotypy may be a promising endophenotype for schizophrenia that, with the appropriate technology, is detectable along the pathway to disease.

Environmental influences in conjunction with a predisposing vulnerability is contemporary psychiatry’s common explanation for the development and evolvement of a psychiatric disorder^23^. Epigenetic changes, such as DNA methylation, provide a mechanism for environmental influences to alter disease risk^24^. Aberrant levels of brain-derived neurotrophic factor (BDNF), a key protein that regulates neuronal development^25^ and neuroplasticity^26^, were shown to be involved in the pathophysiology of stress-related psychiatric outcomes^27^. Varying effects of different stressors on the regulation of BDNF transcripts, including both upregulation and downregulation, have been found in animal models of stress [for review article, see ref. ^28^], and altered BDNF methylation was found in blood samples of stressed humans^29^. Epigenetic modification of BDNF was found in postmortem brains of schizophrenic individuals and in brains of mice with schizophrenia-like behavioral abnormalities born from prenatal stress mice^30^. Several studies have shown alterations in BDNF methylation in blood samples of schizophrenic individuals^31,32^, although the results have been mixed^33,34^. Given the role of BDNF in neuroplasticity^26^, changes in BDNF methylation have been suggested as an important mediator of environmental stress on psychopathology. Therefore, the association between BDNF methylation and suspected neural substrates in psychopathology have been studied. For example, higher BDNF methylation was recently reported to be associated with higher amygdala reactivity to negatively-valanced emotional face processing in healthy individuals^35^. Increased BDNF methylation was also associated with medial prefrontal cortex activity when watching stressful stimuli in violence-related samples^36^. However, there has been limited attention to the role of epigenetic reprogramming of the BDNF gene in relation to the resting-state brain functional network in individuals with schizophrenia.

To determine how schizophrenia emerges, it is crucial to investigate interactions between vulnerability and environmental influences on known altered brain networks related to schizophrenia. Because vulnerability exists as a continuum in the general population^11^, it is important for its analysis to encompass general and clinical individuals and to use psychometrically defined schizotypy as a proxy measure. Because UHR individuals, who are at ~30% risk for overt psychotic disorder within 2–3 years of follow-up^37^, are relatively free from the confounding effects of the chronicity of illness in schizophrenia^38^ and prolonged exposure to antipsychotic medications^39^, they were chosen for this study. Investigating schizotypy in UHR individuals could also provide evidence of schizotypy as an additional strategy to identify “true positive” individuals in the pre-psychotic phase. Thus, the objectives of this study were (1) to investigate changes in resting-state brain network FC in UHR individuals compared with healthy control (HC) individuals and (2) to evaluate the moderating effect of schizotypy on the relationship between BDNF methylation on network FC. Based on previous studies, we hypothesized that changes in network FC in UHR individuals would occur between the DMN and the FPN and that the level of schizotypy would influence the relationship between BDNF methylation and the aberrant network FC across HC and UHR individuals.

## Results

### Demographic and clinical characteristics

Participant characteristics are provided in Table 1. Age did not differ significantly between the HC and UHR groups. Compared to the HC group, the UHR group had a higher percentage of male participants (*χ*^*2*^ = 5.3, *P* = 0.022) and a lower level of education (*χ*^*2*^ = 11.9, *P* = 0.018). SIPS diagnoses of UHR individuals were as follows: Only APS (*n* = 31), only BIPS (*n* = 1), APS and GRDS (*n* = 10), APS and BIPS (*n* = 3), and APS, BIPS, and GRDS combined (*n* = 1). Fifteen UHR individuals were medicated with antipsychotics [chlorpromazine equivalent dose^40^, mean (SD) = 176.1 (115.4)].

**Table 1:** Demographic and clinical characteristics of the study groups.

Variable	HC (*N* = 101)	UHR (*N* = 46)	*P*-value
Sex, male/female, No.	48/53	31/15	0.032
Age, mean (SD), y	21.2 (2.8)	20.9 (4.2)	0.629
Educational level, No^a^			0.018
High school attendances	9	13	
High school graduates	13	8	
College or university undergraduates or dropouts	66	19	
College graduates	4	1	
University graduates	9	5	
Employment status^b^			0.151
Employed	3	2	
Student	76	20	
Unemployed	10	6	
Marital status, single/married	100/1	46/0	0.630
SIPS score, mean (SD)^c^			
Positive symptoms	—	11.7 (4.0)	
Negative symptoms	—	12.5 (5.9)	
Disorganization symptoms	—	3.6 (2.6)	
General symptoms	—	6.8 (4.0)	
Antipsychotic medications			
Naïve/medicated, No.	—	31/15	
Chlorpromazine equivalent dose,^d^ mean (SD), mg/d	—	176.1 (115.4)	

Abbreviations: *HC* healthy controls, *SD* standard deviation, *SIPS* structured interview for prodromal syndromes, *UHR* ultra-high risk for psychosis.

^a^High school, years 10–12; College, years 13–14; University, years 13–16.

^b^Employment status data were available for 89 HC and 28 UHR participants.

^c^SIPS data were available for 44 UHR participants.

^d^Chlorpromazine equivalent dose was derived from Kroken et al.^40^.

### PAS and BDNF DNA methylation

**Fig. 1:**
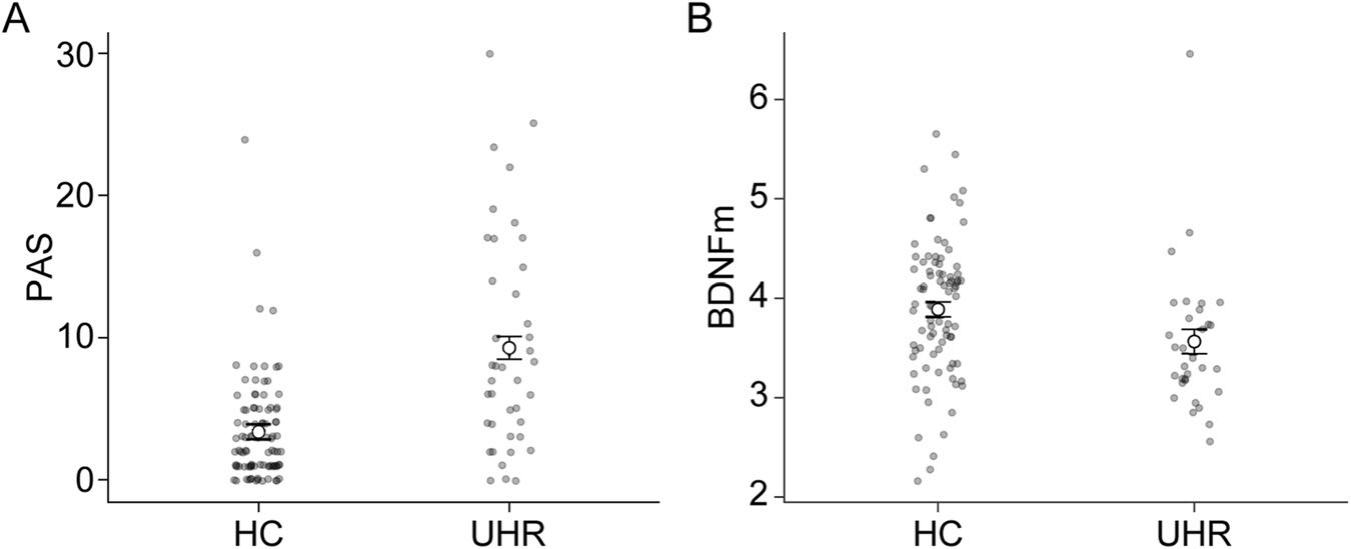
Comparisons of schizotypy and BDNF methylations between HC and UHR. The UHR group showed higher PAS scores (**A**) and lower percentages of BDNF methylation (**B**) than HCs. Abbreviations: BDNFm the percentage of BDNF methylation, HC healthy control, PAS perceptual aberration scale, UHR ultra-high risk for psychosis.

The UHR group had higher PAS scores than the HC group after controlling for age, sex, and level of education [*n*: HC = 93, UHR = 41; mean (SD): HC = 3.4 (3.7), UHR = 9.3 (7.5); *F(1,132)* = 26.2, *P* < 0.001; Fig. 1A]. When comparing HCs, UHR individuals not diagnosed with genetic risk and deterioration syndrome (*n* = 31), and UHR individuals diagnosed with genetic risk and deterioration syndrome (*n* = 10), UHR individuals with genetic risk showed significantly higher PAS scores (*P* < 0.001; Supplementary Fig. 3). Regarding BDNFm, UHR individuals showed lower percentages of BDNF methylation than HCs after controlling for age, sex, and level of education [n: HC = 83, UHR = 32; mean (SD): HC = 3.9 (0.7), UHR = 3.6 (0.7); *F(1,113)* = 4.6, *P* = 0.033; Fig. 1B].

### DMN-FPN network FC

**Fig. 2:**
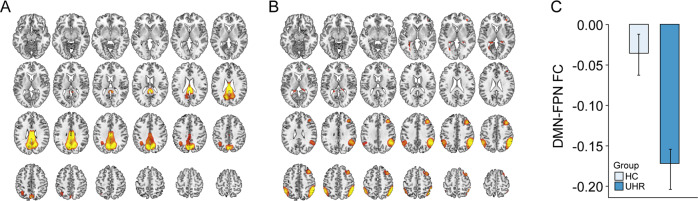
The DMN-FPN network FC differences between HC and UHR. **A** Spatial map of the DMN network. **B** Spatial map of the FPN network. **C** Between-group comparisons of the DMN-FPN network FC. Abbreviations: DMN default-mode network, FC functional connectivity, FPN frontoparietal network, HC healthy control, UHR ultra-high risk for psychosis.

The DMN-FPN network FC (Fig. 2A, B) differed between the HC and UHR groups after controlling for age, sex, and level of education. Compared to HCs, UHR individuals showed reduced DMN-FPN network FC [*F(1,145)* = 9.4, *P* = 0.003; Fig. 2C]. After controlling for age, sex, educational level, and dose of medication in UHR individuals, the DMN-FPN network FC remained reduced in UHR individuals compared to HCs [*F(1,145)* = 5.2, *P* = 0.024].

### Interaction between PAS and DNA Methylation on the DMN-FPN network FC across HC and UHR individuals

**Fig. 3:**
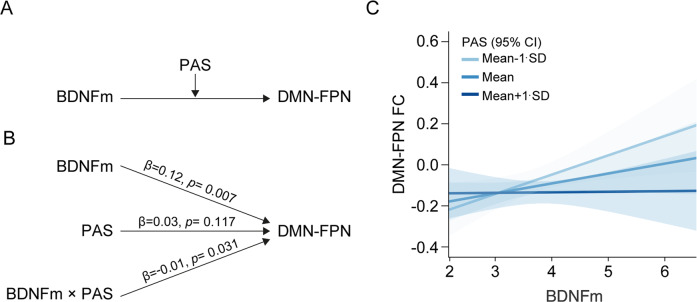
Interaction between PAS and BDNFm on the DMN-FPN network FC. **A** Conceptual and **B** statistical models and **C** the plot showing the simple effects with standard errors of the estimates to visualize the association between BDNF methylation and the DMN-FPN network FC moderated by PAS. Regression coefficients were calculated in a moderation analysis model including age, sex, and years of education as covariates. Abbreviations: BDNFm the percentage of BDNF methylation, DMN default-mode network, FC functional connectivity, FPN frontoparietal network, HC healthy control, PAS perceptual aberration scale, UHR ultra-high risk for psychosis.

PAS scores moderated the relationship between BDNFm and the DMN-FPN network FC after controlling for age, sex, and level of education (*P* = 0.031, *f*^2^ = 0.047, LLCI/ULCI = −0.0193/−0.0009, Fig. 3). When the additional analysis was conducted, including the group as an independent variable in the model, the moderating effect of PAS scores remained (*P* = 0.046). BDNFm had an effect on the DMN-FPN FC when PAS scores were one SD below the mean (*β* = 0.11, *P* = 0.008) and at the mean (*β* = 0.09, *P* = 0.017), but not at one SD above the mean (*β* = 0.03, *P* = 0.319). Therefore, as the level of schizotypy (PAS score) increased, the strength of the positive relationship between BDNFm and the network FC decreased. When the analysis was repeated controlling for age, sex, level of education, and medication dosage, the effect of PAS on the relationship between BDNFm and the DMN-FPN network FC remained (*P* = 0.045).

## Discussion

UHR individuals showed reduced DMN-FPN network FC compared to HCs. Moreover, the DMN-FPN network FC was affected by the interaction between the level of schizotypy and the percentage of BDNF methylation across HC and UHR individuals. To the best of our knowledge, this is the first study to investigate the effect of schizotypy, a proxy of schizophrenia liability, in conjunction with BDNF methylation, which represents environmental exposure, on vantage points of the stress-vulnerability model. Because the schizotypy-BDNF methylation interaction was associated with altered network FC, which was significantly different between HC and UHR individuals, our findings support the widely accepted explanation of the development of schizophrenia by environmental influences in conjunction with predisposing vulnerability.

Consistent with previous studies on schizophrenia [for meta-analysis, see^7^] and UHR individuals^8,9^, the current study demonstrated aberrant coordination between the DMN and the FPN in UHR individuals. The disconnection model with regards to the onset of schizophrenia has been previously reviewed^7,41^. According to this model, there is a disturbance in the maintenance of the integrated self, which may be a core phenomenon of schizophrenia^42^ and may be associated with further enriched risk of overt psychosis conversion in UHR individuals^43^, that is tightly linked to dysconnectivity between the large-scale brain systems responsible for internal thought (DMN), goal-directed regulation (FPN), salience processing (ventral attention network), emotion processing (affective network), and gating information (thalamus network)^7^. The reduced network FC finding in our UHR group supports the disconnection model and confirms the hypo-connectivity between the DMN and the FPN, which reflects an imbalanced coordination between self-related activity and externally focused cognitive activity. Although all the brain networks listed above were not analyzed in this study, the significant difference that we found in the DMN-FPN network FC between the UHR and HC groups supports the rationale to measure the DMN-FPN network FC when evaluating the neurobiological substrates of schizophrenia.

In regards to the DMN-FPN network FC, the novel challenge of this study was to examine the statistical interaction between the level of schizotypy and BDNF methylation to determine whether vulnerability and environmental influence have a joint impact on the underlying neurobiological mechanism of schizophrenia. The vast majority of studies on the interplay between genetic risk and environmental exposure driving schizophrenia have used indirect measures^44^. Likewise, this study used the level of schizotypy as a proxy for the genetic-based liability to schizophrenia and the percentage of methylation of the BDNF promoter to represent environmental influence. As revealed by examining the moderating effect of schizotypy on the association between BDNF methylation and the DMN-FPN network FC in this study, the positive relationship between BDNF methylation and the network FC decreased when the level of schizotypy was high. The additional analysis, including the group as an independent variable in the model, showed that the association was not driven by group differences. The remaining moderating effect of schizotypy after additionally controlling for chlorpromazine equivalent dose eliminated the confounding effects of antipsychotic medications. A remaining question is how epigenetically inhibited BDNF transcription could enhance network FC. One possible explanation is that downregulated BDNF may directly modulate synaptic strength to alter environmental sensitivity^45^. Another possibility is the effect of BDNF on other neurotransmitter systems^46,47^, which in turn modifies brain reactivity. Previous studies on the effect of peripheral DNA methylation on FC changes in schizophrenia have reported inconclusive results across heterogenous target genes [for review article, see ref. ^48^]. In the case of BDNF methylation on brain activity related to schizophrenia, one study^49^ found that BDNF methylation in healthy individuals was associated with prefrontal cortex functional activity and working memory accuracy, which was used as an index of phenotypes relevant to schizophrenia. Our findings and previous research^49^ suggest that BDNF methylation can bridge the environmental exposures to the endophenotypes of schizophrenia; however, caution is required when interpreting hypothetical linkages between BDNF methylation and brain network changes in schizophrenia due to the scarcity research. With this precaution, the moderation effect of schizotypy on the positive association between BDNF methylation and network FC indicates that dampening of this positive association in individuals with high levels of schizophrenia liability may result in reduced DMN-FPN network FC in individuals at high risk for schizophrenia who show elevated levels of schizotypy.

At present, despite extensive studies on BDNF methylation and the development of various psychiatric disorders, changes in methylation have not been used as a specific biomarker for certain psychiatric phenotypes. In addition, most of the changes in BDNF methylation have been very small between clinical samples and healthy controls, a 1.3% difference in individuals with schizophrenia as an example^50^. However, a 9–15% change in BDNF methylation has been suggested as necessary to induce alterations in BDNF transcripts^50^. A recent study also reported no significant difference in mean methylation of BDNF among first-episode psychosis individuals, their unaffected siblings, and healthy controls; however, the study found higher levels of methylation in individuals with childhood trauma independent of diagnosis^34^. The nonspecific association of BDNF methylation with psychiatric disorders and the discordance between the level of BDNF methylation and its protein may be due to other regulators of expression. BDNF methylation itself may reflect regulation of genetic expression instead of direct influence^51^, and stressors may interrupt this gene expression balance^28^. Recent studies on the epigenetic inheritance of DNA methylation^52,53^ also have suggested a novel underlying mechanism of epigenetic regulation. Therefore, a comprehensive analysis of encompassing factors that impact BDNF expression is needed to determine environmental influence both on the level of BDNF methylation and its expression.

The UHR construct has enabled researchers to identify individuals at high risk for the transition to schizophrenia. Because of “false positive” cases in UHR individuals, researchers have tried to use additional strategies to identify “true positive” individuals in the pre-psychotic phase. It remains unclear how schizotypy is positioned in the high-risk research paradigm. However, previous research on schizotypy in the general population^54,55^, genetically high risk individuals^56–58^, and clinically high risk individuals^59–61^ has suggested that schizotypy associates with risk of development of psychosis [for a review, see ref. ^62^]. Our results provide evidence for schizotypy as a proxy for genetic-based vulnerability to schizophrenia, by showing the differences of PAS scores among HC, UHR individuals without genetic risks, and UHR individuals with genetic risks. Thus, this study suggests that measuring schizotypy could identify individuals with ‘*true*’ susceptibility for aberrant network FC when combined with epigenetic changes.

This study has several limitations. First, the cross-sectional data limits understanding of any causal relationships. The medium sample size of UHR individuals did not allow for generalization of our findings, such as the range of percentages of methylation of the BDNF promoter. It was also restricted to analyses of the relationships between the variables and psychotic transition. Therefore, further prospective studies with larger numbers of UHR individuals, who are thoroughly followed-up to observe clinical outcomes and assessed by using interviewer-rated anomalous self-experience measures, such as the Examination of Anomalous Self-Experience^63^, are needed in the near future. BDNF methylation was measured to reflect BDNF gene regulation or epigenetic mechanisms as directly as possible. However, given the discordance between the level of BDNF methylation and its protein^51^, analyzing the mRNA or protein levels of BDNF could provide detailed information about BDNF gene expression and its relationship with FC in further studies. Lastly, some UHR participants were taking antipsychotic medications. Although the use of covariates in the analysis cannot completely rule out potential confounding effects, this study yielded identical results when the analyses were repeated controlling for dosage of antipsychotic medications in addition to age, sex, and level of education. Thus, the effect of antipsychotic medications is unlikely to change the overall findings in this study.

In conclusion, the main findings of this study support the hypothesis that the impact of schizotypy on the candidate network FC, the putative underlying neural substrate of schizophrenia, is due to interactions with epigenetic changes that reflect environmental influence. By demonstrating the moderating role of schizotypy on the association between BDNF methylation and changes in the DMN-FPN network FC, this study provides pivotal neurobiological data substantiating the stress-vulnerability model of developing schizophrenia.

## Methods

### Participants

Participants initially included 101 healthy young adults and 46 UHR individuals. Participants were excluded from some analyses if they had missing or incomplete data. Healthy individuals were recruited via online advertisements, and UHR individuals were enrolled from a specialized UHR clinic as part of the Green Program for Recognition and Prevention of Early Psychosis (GRAPE) project at Severance Hospital in Seoul, Republic of Korea. All participants were screened for psychiatric illnesses using the Structured Clinical Interview for Diagnostic and Statistical Manual of Mental Disorders, Fourth edition^64,65^, and individuals with any current or past history of major psychiatric disorders were excluded from the HC group. UHR participants were diagnosed using the Structured Interview for Prodromal Syndromes [SIPS; ref. ^66^]; therefore, each UHR participant met one or more of the three diagnoses: attenuated positive symptom syndrome (APS), brief intermittent psychotic symptom syndrome (BIPS), or genetic risk and deterioration syndrome (GRDS). Further details on the GRAPE project have been described previously^67,68^. This study was reviewed and approved by the Institutional Review Board at Severance Hospital, and the study was performed in accordance with the Declaration of Helsinki. All of the participants and the guardians of the participants (if younger than 18 years old) provided written informed consent. A study flow chart summarizing the number of individuals in the analyses is presented in Supplementary Fig. 1.

### Psychometric measure of schizotypy

Because previous researchers (Rado, 1960; Meehl, 1964) described a key component of schizotypic phenotypes as an aberrant awareness of one’s body, a well-known psychometric tool, the Perceptual Aberration Scale [PAS; ref. ^69^] has been used to index schizophrenia liability. The PAS contains 35 items to assess psychotic-like perceptual experiences, which includes bodily discontinuities and unusual scenery experiences (e.g., “I have felt that something outside my body was a part of my body”). PAS-identified persons with high levels of schizotypy were found to be similar to schizophrenics with regards to various psychological, cognitive, and physiological factors^15^. Moreover, genomic association of the PAS score with a single nucleotide polymorphism of interest to schizophrenia research^70^ provided genetic evidence that the etiology of schizophrenia involves perceptual aberrations. Perceptual aberrations also associated with simpler psychological processes, such as exteroceptive and proprioceptive tasks^71^, rather than complex constructs; therefore, reducing heterogeneity by focusing on PAS could reveal underlying biological states^15^. Thus, to evaluate their levels of schizotypy, all participants were asked to complete the PAS; however, PAS data for 8 HC and 5 UHR individuals were missing.

### Epigenotyping procedures

Genomic DNA was extracted from 83 HC and 32 UHR individuals [peripheral whole blood (*n*; HC = 76, UHR = 25); saliva (*n*; HC = 7, UHR = 7)]. A CpG-rich region of the BDNF promoter, including seven CpG sites between −694 and −577 relative to the transcriptional start site, was analyzed [GenBank accession number: JX848620^72^; Supplementary Fig. 1]. This region was studied in Korean individuals^72,73^ and differential methylation of the analogous region in rat BDNF was associated with BDNF mRNA expression^74^. DNA methylation status was determined by Macrogen, Inc. (Seoul, Republic of Korea) using standard procedures (for the detailed protocol, see Supplementary Methods). Average values of the percent methylation at the seven CpG sites of the BDNF promoter (BDNFm) were used in the analyses.

### Magnetic resonance imaging (MRI) data acquisition

MRI data were acquired using a 3T scanner (Intera Achieva, Philips Medical Systems, Best, The Netherlands). Participants were instructed to rest in the scanner with their eyes closed and to stay still and quiet without sleeping or focusing on any specific thought. T2*-weighted gradient echo-planar imaging was used: repetition time (TR) = 2000 ms, echo time (TE) = 30 ms, flip angle = 90°, 31 interleaved slices, matrix size = 80 × 80, voxel size = 2.75 × 2.75 × 4 mm^3^, field-of-view (FOV) = 220 mm. High-resolution structural T1-weighted images were acquired using the turbo field echo sequence with the following settings: TR = 9.7 ms, TE = 4.6 ms, flip angle = 8°, 180 slices, matrix = 256 × 256, voxel size = 0.859 × 0.859 × 1.2 mm^3^, FOV = 220 mm.

### Image preprocessing

Functional MRI (fMRI) data were analyzed using Statistical Parametric Mapping 12 software (Wellcome Trust Centre for Neuroimaging, London, UK) and Functional Connectivity toolbox version 19b (CONN; McGovern Institute for Brain Research, Massachusetts Institute of Technology, Cambridge, MA, USA)^75^ in MATLAB (Mathworks Inc., Natick, MA, USA). The first five volumes of the functional datasets from each participant were discarded to ensure signal stabilization. Using CONN’s default preprocessing pipeline, the remaining images underwent standard preprocessing steps: functional realignment and unwarp, functional and structural center to (0, 0, 0) coordinates, slice-timing correction, identification of outliers (global mean *z*-threshold < 3, motion threshold < 1 mm) with the Artifact Detection Tool, segmentation, normalization to the standard Montreal Neurological Institute space, and functional smoothing (spatial convolution with a 6 mm full-width Gaussian kernel at half-maximum). The images were resampled to 2 × 2 × 2 mm^3^ voxels. After preprocessing, fMRI data were denoised using CONN’s default denoising pipeline: linear regression of potential confounding effects (motion-related artifacts, noise components from white matter and cerebrospinal areas, scrubbing), temporal band-pass filtering (0.008–0.09 Hz), and linear detrending.

### Group-independent component analysis

Group-independent component analysis [ICA; ref. ^76^] was performed to identify the intrinsic functional organization of the brain as follows: group-level dimensionality reduction using principal component analysis, estimation of spatially-independent components (ICs) using the FastICA algorithm, and back-reconstruction of individual-level ICs using dual regression. The current study constrained the number of ICs to 30, which is suitable for a low-dimensional ICA decomposition^77^. Group-averaged ICs were inspected by two experienced investigators (SKA and HYP) to identify networks involving the DMN and FPN. ICs were labelled based upon agreement between experts. Moreover, to better characterize each network, a meta-analytic decoding of ICs was also performed using NeuroSynth (http://neurosynth.org^78^) (Supplementary Table S1). Calculation of the correlation coefficient between the time courses of each spatial component estimated the network FC between the DMN and the FPN. FC values for the DMN-FPN of each participant were extracted for further analysis.

### Statistical analysis

Demographic variables were compared using the independent *t*-test and chi-square test. For comparing PAS scores and BDNFm between groups, we performed analyses of covariance (ANCOVA) using the age, sex, and level of education as covariates. Square root transformation was applied to the PAS scores to produce a normal distribution of responses; therefore, all variables had skewness levels less than 1.0, which was acceptable for ANCOVA.

When determining a statistically significant difference in the FC of the DMN-FPN between HC and UHR individuals, ANCOVA was conducted controlling for age, sex, and level of education.

To determine whether schizotypy moderated the relationship between BDNF methylation and the DMN-FPN FC network, the effects of PAS scores and BDNFm on the DMN-FPN FC were analyzed using the simple moderation model in PROCESS macro in SPSS^79^ with age, sex, and level of education as covariates. Because the results might be driven by group differences, the additional analysis was conducted, including using the group as an independent variable in the model. To control for possible confounding effects of antipsychotic medications in UHR individuals, the analysis was also repeated by controlling for the chlorpromazine equivalent dose, as well as age, sex, and level of education. For all analyses, *P* < 0.05 was considered statistically significant.

## Supplementary information

Online supplementary material

## Data Availability

The data that support the findings of this study are available from the corresponding author upon reasonable request.
