# Supplementary material for: The stress-vulnerability model on the path to schizophrenia: Interaction between BDNF methylation and schizotypy on the resting-state brain network
Source: Schizophrenia (Heidelb). 2022 May 6;8(1):49. doi: 10.1038/s41537-022-00258-4 (PMC9261098; doi:10.1038/s41537-022-00258-4)
Supplement: Supplementary file 1 — Online supplementary material [file 41537_2022_258_MOESM1_ESM.docx]

**Online Supplementary Material**

**Supplementary method**

*Bisulfite pyrosequencing DNA methylation analysis*

Genomic DNA was prepared from peripheral whole blood or saliva using standard techniques for DNA methylation analysis by Macrogen, Inc. (Seoul, Republic of Korea). Total genomic DNA was prepared from leukocytes using the MG Clinic Genomic DNA Extraction SV miniprep (Doctor Protein Inc., Republic of Korea). DNA purity (A280/260) and concentration (ng/µL) was evaluated using 2 μL nanodrops with an Epoch microplate spectrophotometer (Bio Tek, Winooski, VT., USA). Genomic DNA was bisulfite-treated using the EpiTect Fast DNA Bisulfite Kit (Qiagen, Hilden, Germany). Pyrosequencing Assay Design 2.0 (Qiagen) was used to design the polymerase chain reaction (PCR) assay and sequencing primers. PCR amplification was conducted using a Pyromark PCR kit (Qiagen) and the following program: denaturation at 95°C for 15 min; followed by 45 cycles at 94°C, 56°C, and 72°C, each for 30 s; followed by a final extension cycle at 72°C for 10 min. Amplified PCR products were pyrosequenced using PyroMark Q48 Autoprep software (Qiagen).

**Supplementary Figure 1.** Study flow chart.

**
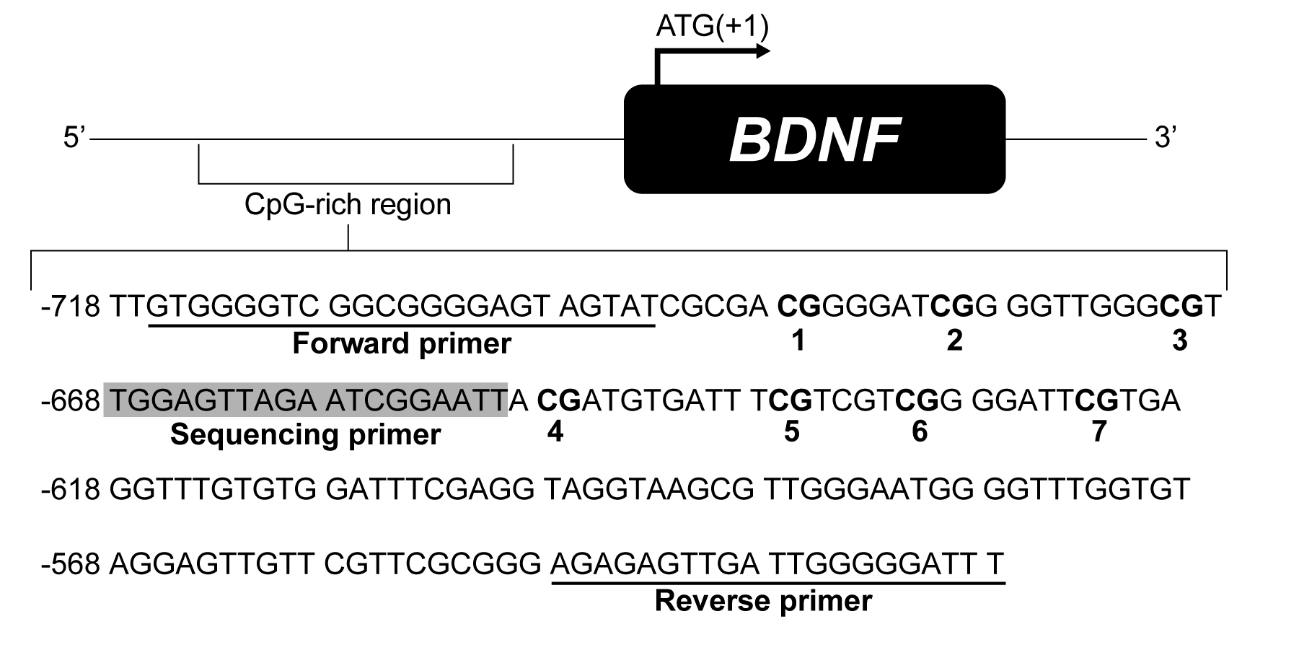
**

**Supplementary Figure 2.** Seven CpG-rich regions of the BDNF promoter were pyrosequenced.


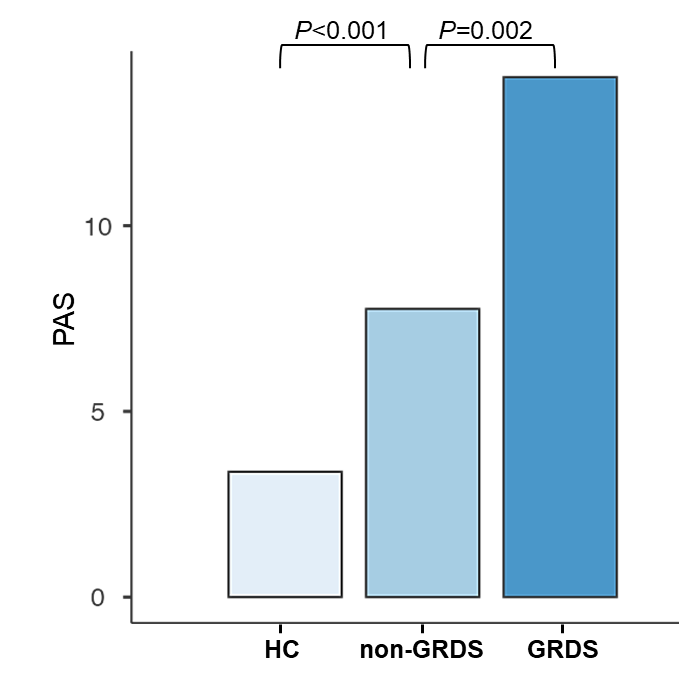


**Supplementary Figure 3.** Comparisons of schizotypy among HCs (n = 93), UHR individuals (n=31) not diagnosed with genetic risk and deterioration syndrome (non-GRDS), and UHR individuals (n=10) diagnosed with genetic risk and deterioration syndrome (GRDS) (*P*<0.001), controlling for age, sex, and education. The GRDS group showed significantly higher PAS scores than the HC or non-GRDS group.

Abbreviations: HC, healthy control; GRDS, UHR individuals diagnosed with genetic risk and deterioration syndrome; non-GRDS, UHR individuals not diagnosed with genetic risk and deterioration syndrome; PAS, perceptual aberration scale; UHR, ultra-high risk for psychosis.

**Supplementary Table 1.** Meta-analytic decoding of the intrinsic functional networks involving the DMN and FPN using NeuroSynth (http://neurosynth.org).^1^ The highest 20 terms ranked by correlation strength are listed.

| DMN | |  | FPN | |
| --- | --- | --- | --- | --- |
| Terms | Correlation (similarity) |  | Terms | Correlation (similarity) |
| Precuneus | 0.330 |  | Inferior parietal | 0.320 |
| Posterior cingulate | 0.324 |  | Parietal | 0.174 |
| Precuneus posterior | 0.217 |  | Angular | 0.155 |
| Cortex precuneus | 0.196 |  | Parietal cortex | 0.146 |
| Posterior | 0.191 |  | Angular gyrus | 0.132 |
| PCC | 0.190 |  | Frontoparietal | 0.124 |
| Cingulate | 0.157 |  | IPL | 0.119 |
| Midline | 0.140 |  | Working memory | 0.112 |
| Memory retrieval | 0.136 |  | Working | 0.112 |
| Cortex PCC | 0.130 |  | Parietal lobule | 0.110 |
| retrieval | 0.126 |  | Dorsolateral prefrontal | 0.098 |
| Cingulate cortex | 0.119 |  | Hippocampal | 0.098 |
| Recognition memory | 0.105 |  | Calculation | 0.097 |
| Recollection | 0.097 |  | Fronto | 0.096 |
| Default mode | 0.094 |  | MTL | 0.096 |
| Default | 0.093 |  | Temporal lobe | 0.096 |
| Retrieved | 0.088 |  | Working memory | 0.095 |
| Cuneus | 0.081 |  | MTL | 0.095 |
| Cortex posterior | 0.079 |  | Hippocampus | 0.092 |
| Theory mind | 0.077 |  | Medial temporal | 0.091 |

Abbreviations: DMN, default-mode network; FPN, frontoparietal network.

**References**

**1.** Yarkoni T, Poldrack RA, Nichols TE, Van Essen DC, Wager TD. Large-scale automated synthesis of human functional neuroimaging data. *Nat Methods* Jun 26 2011;8(8):665-670.
